# The Difficulty of Detecting Occult Metastases in Patients with Potentially Resectable Pancreatic Cancer: Development and External Validation of a Preoperative Prediction Model

**DOI:** 10.3390/jcm13061679

**Published:** 2024-03-14

**Authors:** Marieke Walma, Laura Maggino, F. Jasmijn Smits, Alicia S. Borggreve, Lois A. Daamen, Vincent P. Groot, Fabio Casciani, Vincent E. de Meijer, Frank J. Wessels, George P. van der Schelling, Vincent B. Nieuwenhuijs, Koop Bosscha, Erwin van der Harst, Ronald van Dam, Mike S. Liem, Sebastiaan Festen, Martijn W. J. Stommel, Daphne Roos, Fennie Wit, Ignace H. de Hingh, Bert A. Bonsing, Olivier R. Busch, Bas Groot Koerkamp, Geert Kazemier, Marc G. Besselink, Roberto Salvia, Giuseppe Malleo, I. Quintus Molenaar, Hjalmar C. van Santvoort

**Affiliations:** 1 Department of Surgery, Regional Academic Cancer Center Utrecht, University Medical Center Utrecht and St. Antonius Hospital, University Utrecht, 3508 GA Utrecht, The Netherlandsi.q.molenaar@umcutrecht.nl (I.Q.M.);; 2 Department of Surgery, Cancer Center Amsterdam, Amsterdam UMC, University of Amsterdam, 1105 AZ Amsterdam, The Netherlands; o.r.busch@amsterdamumc.nl (O.R.B.);; 3 The Pancreas Institute, University of Verona Hospital Trust, 37134 Verona, Italyfabio.casciani@univr.it (F.C.); giuseppe.malleo@aovr.veneto.it (G.M.); 4 Department of Surgery, University Medical Center Groningen, University of Groningen, 9713 GZ Groningen, The Netherlands; v.e.de.meijer@umcg.nl; 5 Department of Radiology, Regional Academic Cancer Center Utrecht, University Medical Center Utrecht and St. Antonius Hospital, University Utrecht, 3508 GA Utrecht, The Netherlands; 6 Department of Surgery, Amphia Hospital Breda, 4818 CK Breda, The Netherlands; 7 Department of Surgery, Isala Hospital, 8025 AB Zwolle, The Netherlands; 8 Department of Surgery, Jeroen Bosch Hospital, 5223 GZ ‘s-Hertogenbosch, The Netherlands; 9 Department of Surgery, Maasstad Hospital, 3079 DZ Rotterdam, The Netherlands; harste@maasstadziekenhuis.nl; 10 Department of Surgery, Maastricht University Medical Center, 6229 HX Maastricht, The Netherlands; r.van.dam@mumc.nl; 11 Department of Surgery, Medical Spectrum Twente, 7511 HN Enschede, The Netherlands; 12 Department of Surgery, Onze Lieve Vrouwe Gasthuis, 1091 AC Amsterdam, The Netherlands; s.festen@olvg.nl; 13 Department of Surgery, Radboud University Medical Center, 6525 GA Nijmegen, The Netherlands; 14 Department of Surgery, Reiner de Graaf Hospital, 2625 AD Delft, The Netherlands; 15 Department of Surgery, Tjongerschans Hospital, 8441 PW Heerenveen, The Netherlands; 16 Department of Surgery, Catharina Hospital, 5623 EJ Eindhoven, The Netherlands; 17 Department of Surgery, Leiden University Medical Center, 2333 ZA Leiden, The Netherlands; 18 Department of Surgery, Erasmus Medical Center, 3015 GD Rotterdam, The Netherlands

**Keywords:** pancreatic cancer, occult metastases, prediction model

## Abstract

Occult metastases are detected in 10–15% of patients during exploratory laparotomy for pancreatic cancer. This study developed and externally validated a model to predict occult metastases in patients with potentially resectable pancreatic cancer. Model development was performed within the Dutch Pancreatic Cancer Audit, including all patients operated for pancreatic cancer (January 2013–December 2017). Multivariable logistic regression analysis based on the Akaike Information Criteria was performed with intraoperative pathologically proven metastases as the outcome. The model was externally validated with a cohort from the University Hospital of Verona (January 2013–December 2017). For model development, 2262 patients were included of whom 235 (10%) had occult metastases, located in the liver (*n* = 143, 61%), peritoneum (*n* = 73, 31%), or both (*n* = 19, 8%). The model included age (OR 1.02, 95% CI 1.00–1.03), BMI (OR 0.96, 95% CI 0.93–0.99), preoperative nutritional support (OR 1.73, 95% CI 1.01–2.74), tumor diameter (OR 1.60, 95% CI 1.04–2.45), tumor composition (solid vs. cystic) (OR 2.33, 95% CI 1.20–4.35), and indeterminate lesions on preoperative imaging (OR 4.01, 95% CI 2.16–7.43). External validation showed poor discrimination with a C-statistic of 0.56. Although some predictor variables were significantly associated with occult metastases, the model performed insufficiently at external validation.

## 1. Introduction

In Western Europe, pancreatic cancer has an incidence of 8.4 per 100,000 inhabitants and is estimated to become the second leading cause of cancer-related death in the near future [1,2]. The majority of patients with pancreatic cancer have an advanced stage of disease at diagnosis, with only 10–20% qualifying for resection [3,4].

Current routine investigations for preoperative staging include a multidetector computed tomography (MDCT) using a dual-phase pancreatic protocol [5]. The accuracy of MDCT in determining resectability is 85–95% and the main reason for unresectability during exploratory laparotomy is the presence of distant metastases that were not detected on preoperative MDCT [5,6]. The reported incidence of occult distant metastases from recent studies is approximately 10–15%, of which the majority are located in the liver [6,7]. Given the dismal prognosis of patients with metastatic pancreatic cancer, together with the possible delay in the start of systemic treatment, it is important to avoid a futile laparotomy whenever possible. 

A potential valuable diagnostic tool to avoid unnecessary laparotomy is a staging laparoscopy to identify peritoneal or liver metastases. However, preoperative cross-sectional imaging resolutions have steadily improved over the years, so that routine staging laparoscopy is still controversial [8]. Nonetheless, a subset of patients with a high risk for occult metastatic disease might benefit from staging laparoscopy. The National Comprehensive Cancer Network (NCCN) guidelines define patients with borderline resectable disease, markedly elevated serum CA19-9 levels, large primary tumors, or large regional lymph nodes as high risk for occult metastases [8]. The guideline advises to consider a staging laparoscopy in those patients. However, cutoff values or a risk model are not available to assist decision making on whether to perform a staging laparoscopy, and the practice differs between hospitals. 

This study aimed to develop and externally validate a preoperative prediction model for occult distant metastases in patients with potentially resectable or borderline resectable pancreatic cancer. 

## 2. Materials and Methods

This study was performed in accordance with the TRIPOD guidelines for the development and validation of multivariable prediction models [9]. Data from the Dutch Pancreatic Cancer Audit (DPCA) were used and a scientific committee governing these data reviewed the study proposal [10]. This prospective registry monitoring quality of care is mandatory for all 18 Dutch centers performing pancreatic surgery and has demonstrated over 90% case ascertainment and over 95% data accuracy [11]. Since the data provided to the study team were anonymized, the need for informed consent was waived. 

### 2.1. Study Population

For the development cohort, all patients with a suspected pancreatic adenocarcinoma, who underwent surgery with intent for resection between January 2013 and December 2017 in the Netherlands, were included from the DPCA. Patients with neuroendocrine tumors were excluded as well as patients younger than 18 years, patients with MDCT imaging more than 6 weeks before surgery, and patients with missing data regarding preoperative imaging or primary outcome. Patients who received neoadjuvant treatment were excluded because it was only given within a clinical trial with a protocolled staging laparoscopy before the start of neoadjuvant therapy [12]. This was less than five percent of the total cohort. Standard work-up for suspected pancreatic cancer included a minimum of an MDCT with a 3 mm slice interval according to a biphasic protocol consisting of an arterial phase and a portal phase (35–40 and 60–70 s after intravenous contrast injection, respectively) [13]. Magnetic resonance imaging (MRI) and/or positron emission tomography computed tomography (PET-CT) were performed on an individual basis and according to local preferences after a multidisciplinary evaluation. The choice of a staging laparoscopy before exploratory laparotomy was at the discretion of the surgeon.

External validation was performed in a prospectively maintained institutional database from a high-volume pancreatic center: the University of Verona Hospital Trust (Verona, Italy; cohort 2013–2017). This cohort included all consecutive treatment-naive patients with pancreatic ductal adenocarcinoma undergoing an exploratory laparotomy or laparoscopy with intent for resection. Similar to the developmental cohort, the standard preoperative work-up always included a 3 mm sliced MDCT using a triphasic pancreatic protocol. In patients deemed to be at high risk for distant metastases (e.g., elevated Ca 19.9 levels, suspicious lesions on MDCT), MRI, PET-CT, and/or staging laparoscopy were recommended by the multidisciplinary team and at the discretion of the treating surgeon. Only patients with available MDCT imaging within 6 weeks before surgery were included. Data were extracted from the institutional database after anonymization and used in compliance with the Institutional Review Board approval for retrospective protocols (PAD-R 1101CESC).

### 2.2. Definitions, Outcome, and Predictors

The primary outcome was defined as pathologically proven liver, peritoneal, or omental metastases during exploratory laparotomy or staging laparoscopy. Potential predictor variables were selected based on a literature search and clinical reasoning. Clinical predictor variables included the following: age, sex, body mass index (BMI), comorbidity, Eastern Cooperative Oncology Group (ECOG) performance status at diagnosis (class 0, 1, and ≥2), weight loss (>1 kg and unintentional), preoperative nutritional support with tube feeding or total parenteral nutrition, serum CA19-9 levels (highest baseline during preoperative period in kU/L), and biliary drainage. Candidate radiographic predictors were the following: tumor location (uncinated process/head, body/tail), biggest tumor diameter, tumor composition (i.e., predominantly solid versus cystic), suspicion of regional lymph node metastases, vascular involvement, T-stage ≥ T3 (according to the American Joint Committee on Cancer TNM classification, 7th edition [14]), and indeterminate lesions on computed tomography (CT) scan and/or MRI. Regional lymph nodes were scored as suspected when the short axis was above 10 mm in diameter [15]. Indeterminate lesions were defined as subcentimetric or aspecific liver or peritoneal lesions on imaging, which could not be definitively characterized or excluded as metastases with further work-up. Other covariables taken into consideration were time from first presentation to surgery and time from MDCT to surgery.

### 2.3. Model Development and Data Analysis

Data were presented as mean with standard deviation (SD), or median with interquartile range when appropriate for continuous data and counts with percentages for categorical data. Expecting an event rate of at least 8% and using the ‘1 to 10 rule of thumb’, a sample size of at least 2250 patients would have been needed to achieve a stable prediction model with 18 candidate predictors [7,16]. Missing variable analysis showed no monotonicity suggesting a random pattern of missing data. Missing data were imputed using multiple imputation (Multiple Imputation by Chained Equations, 20 imputed datasets with a maximum number of 5 iterations for each imputation) [17,18]. Continuous variables were log transformed and systematically tested to explore non-linearity with the primary outcome. Only tumor size turned out to perform better when transformed. In each imputed dataset, the full multivariable logistic regression model including the variables was fitted as described, with occult metastases as the outcome variable. Subsequently, stepwise backward selection based on the Akaike Information Criterion was used to select the relevant variables [19]. This resulted in 20 sets of variables being selected in the 20 imputed sets based on the Akaike Information Criterion. Variable selection for the multivariable logistic regression model took place using the majority rule; that is, the variable was retained within the model when the variable was appearing in at least 50% of the imputation sets [20]. Further stepwise backward selection was based on the likelihood ratio test. The final multivariable logistic regression model was fitted with these selected predictors in each imputation set, and model coefficients were pooled using Rubin’s rules [21]. The discriminatory ability of the model was evaluated by the area under the receiver operating characteristic (ROC) curve in the development set and the external validation set, resulting in the C-statistic. Model calibration of the final model was evaluated by visual inspection of the model calibration plot.

All statistical analyses were performed using R 3.1.2 open-source software (‘mice’, ‘MASS’, ‘pROC’, and ‘rms’ packages, http://www.R-project.org (accessed on 26 October 2021). A *p*-value of <0.05 was considered statistically significant.

## 3. Results

In total, 2925 patients who underwent an exploratory laparotomy or laparoscopy with the intention for pancreatic resection were included for model design and external validation. Baseline characteristics are given in Table 1. Within the development cohort (n = 2262), the mean age was 66 years (SD ± 10), 88% (n = 1836) had an ECOG performance score of 0–1, and the median highest preoperative serum CA19-9 was 110 kU/L (IQR 20–490). Tumors were mostly located in the pancreatic head or uncinate process (n = 1251, 81%). Furthermore, 3% (n = 57) of patients had indeterminate lesions on imaging, which could not be characterized or excluded as metastases with further work-up. A total of 282 patients (13%) underwent staging laparoscopy. Laparoscopy was conducted in the same session as the exploratory laparotomy with intention for resection in 88% of those cases (n = 231). 

In total, 10% (n = 235) of patients were diagnosed with occult distant metastases. In 60 of these patients (26%), a staging laparoscopy was performed. Staging laparoscopy resulted in the detection of occult metastasis in 42/60 patients (70%). The majority of occult distant metastases were found during exploratory laparotomy not preceded by staging laparoscopy (n = 175, 74% of all patients with occult metastases). Most metastases were located in the liver (n = 143, 61%), followed by peritoneal lesions (n = 73, 31%) or both (n = 19, 8%) (Table 2). In patients who received an MRI preoperatively, the frequency of occult metastases was 11% (64/610).

Baseline characteristics of the validation cohort (*n* = 663) are shown in Table 1. Patients in the validation cohort had less comorbidity (54% vs. 80%), had less often weight loss (47% vs. 74%), and received less often preoperative nutritional support (1% vs. 7%). In the development cohort, a higher rate of vascular involvement was observed (35% vs. 8%). The incidence of occult metastases within the validation cohort was 9% (*n* = 60), with 65% (*n* = 39) located in the liver, 25% (*n* = 15) located in the peritoneum, and 10% (*n* = 6) located in both (Table 2).

### Model Development and Performance

Univariable analyses identified age, ECOG PS, weight loss, preoperative nutritional support with tube feeding or total parenteral nutrition, CA19-9, tumor diameter, a cystic tumor composition, vascular involvement, ≥T3 tumor, indeterminate lesions on imaging, and the number of weeks from the first consultation to surgery as variables that were significantly associated with occult metastases (Table 3). The final multivariable model included the following predictors of distant metastases during exploratory surgery (Table 4): higher age (OR 1.02, 95% CI 1.00–1.03), lower BMI (OR 0.96, 95% CI 0.93–0.99), preoperative nutritional support (OR 1.73 95% CI 1.010–2.74), larger tumor diameter (OR 1.60, 95% CI 1.04–2.45), solid tumor composition (versus cystic; OR 2.33, 95% CI 1.20–4.35), and indeterminate liver or peritoneal lesions on imaging (OR 4.01, 95% CI 2.16–7.43).

**Figure 1:**
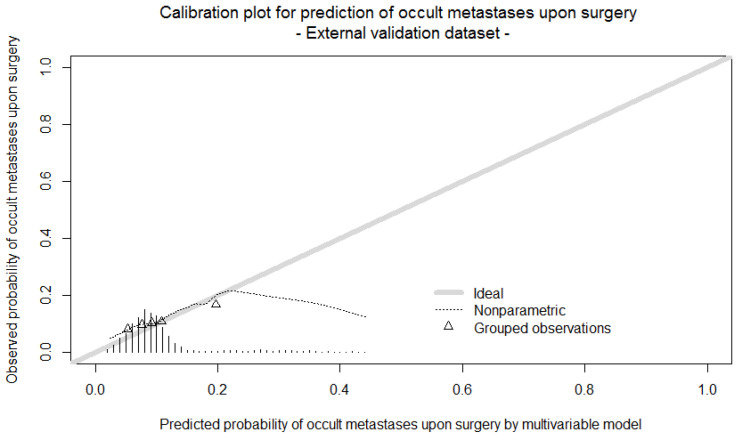
Calibration plot of external validation. Triangles indicate the observed frequencies by deciles of predicted probability.

The model had a moderate discriminatory ability in the development cohort with a C-statistic of 0.65. External validation, using the Verona data cohort, demonstrated a poor discriminatory ability with a C-statistic of 0.56, and a poor calibration upon visual inspection (Figure 1).

## 4. Discussion

In this study, 10% of patients with potentially resectable and borderline resectable pancreatic cancer had occult metastases during exploratory laparotomy or staging laparoscopy. Higher age, lower BMI, preoperative nutritional support with tube feeding or parenteral nutrition, a solid tumor composition (versus cystic), a larger tumor diameter, and indeterminate lesions on preoperative imaging were identified as predictors for the presence of occult metastases during surgery. Although these predictor variables were significantly associated with occult metastases, the discrimination ability of the prediction model was insufficient after external validation within the Verona dataset.

The most frequently identified predictors from other studies that performed multivariable analyses to predict occult metastases were serum CA19-9 and tumor size [7,22,23,24,25,26,27,28,29]. Within the current study, tumor size was an independent predictor for occult metastases, but CA19-9 was only associated with occult metastases in the univariable analysis. Since only data on the highest serum value during the preoperative period were available, without correlation with bilirubin levels or biliary drainage, CA19-9 should not be ruled out as a relevant predictor for future studies. Other biomarkers, such as CA125 or circulating tumor DNA, have been mentioned in studies as potential predictive biomarkers for the detection of occult metastases [30,31]. Unfortunately, these often experimental biomarkers are not routinely assessed preoperatively in the Netherlands and were thus not available in our dataset. However, future studies should try adding these and other novel biomarkers to increase the performance and discrimination of the model.

While preoperative nutritional support and BMI have not been studied before in relation to the presence of occult metastases, previous studies did report (back) pain, jaundice, and weight loss as potential predictors for occult metastases [7,28,29]. Moreover, sarcopenia has been described as a predictor for worse oncological outcomes and a high tumor burden in different types of cancer [32]. Data on myopenia or myosteatosis were not available in our dataset, but it is suggested that these factors indicate a worse physical condition. Future studies should try adding these variables to increase the performance of the model. In addition to being a surrogate marker for physical condition, a lower BMI can cause lower visceral adipose tissue which can impede the evaluation of staging CT scans and thereby lowering the sensitivity for distant metastases [33]. Concerning the radiological composition of the tumor, an explanation for the association with occult metastases can be that adenocarcinomas derived from cystic intraductal papillary mucinous neoplasms (IPMNs) are diagnosed in earlier stages [34]. Some studies also suggest a better biological behavior when compared to conventional pancreatic adenocarcinoma. The association of cystic tumor composition with less occult metastases in the current study supports this hypothesis.

An explanation for the disappointing discrimination and calibration within the Verona dataset might have been the case mix and differences in preoperative management between the two centers [35]. For example, patients from the Verona cohort less frequently received nutritional support compared to the DPCA cohort (Table 1). Moreover, patients with vascular involvement often received neoadjuvant treatment within the Verona cohort, and those were excluded from the current analysis. Another reasonable explanation is that preoperative patient and tumor characteristics are simply not sufficient enough to predict outcomes. Tumor biology is difficult to measure and especially for pancreatic cancer, until now, clinically useful biomarkers are lacking [36].

Overall, in this study 10% of all patients had occult metastases, which were identified during laparoscopy in 70% of patients in whom a staging laparoscopy was performed. This is in line with the PREOPANC 1 trial, where the yield of a staging laparoscopy in all patients in the experimental arm was 11% [12]. In the absence of a solid externally validated risk model, one might argue that a staging laparoscopy should be performed in all patients with potentially resectable or borderline resectable pancreatic cancer. It can prevent the morbidity and possible delay in systemic treatment that accompanies an unnecessary laparotomy in a patient’s final stage of life [37]. Although it was no primary outcome in this study, according to published studies, performing a staging laparoscopy in all patients might prevent a futile laparotomy in around 1 out of 10 to 20 patients [12].

The lack of a reliable and validated risk model for occult metastases is reflected in the current guidelines. A consensus document from the Americas Hepato-Pancreato-Biliary Association includes equivocal findings on imaging, together with a tumor size above 3 cm, CA19-9 above 100 kU/L, and body/tail lesions as predictors on the basis upon which patients should be selected for staging laparoscopy [37]. The NCCN guidelines define markedly elevated serum CA19-9 levels without further specification, borderline resectable disease, large primary tumors, or large regional lymph nodes as high risk for occult metastases as a guide to perform a staging laparoscopy [8,38]. The current study cannot confirm these strategies and can only conclude that occult metastases are difficult to predict.

An important subject of ongoing improvement is the field of imaging techniques. Within the current study, patients that underwent MRI had a similar frequency of occult metastases (11%). However, with enhancing technologies, it is likely that the preoperative risk assessment could be improved with, for example, improved DWI sequences and hepatobiliary contrast series during magnetic resonance investigation (MRI) or alternative tracers in (PET-) CT imaging [39,40,41]. Furthermore, within the current era of emerging neoadjuvant therapies, future studies should investigate the use of staging laparoscopy in patients after neoadjuvant therapy as well. Lastly, new biomarkers are extensively studied. For example, CA125, circulating tumor cells, or tumor DNA or RNA could be of use when predicting occult metastases and should be included in future models in addition to preoperative patient and tumor characteristics [30,31,42]. A recent pilot study described a phenotype circulating tumor cell (CTC)-based blood test with a 100% sensitivity and 89% specificity for metastases in pancreatic cancer. Although the sample size was small and the test needs a specific device to harvest CTCs, these results should be regarded as promising [43].

The strengths of this study include the sample size, extent, and multicenter character of the DPCA dataset, which allowed a proper statistical analysis. Moreover, this study was the first to attempt a broad validation of a predictive model for occult metastases in patients with pancreatic cancer, or at least the first to publish the negative results. It is of great importance to also publish validation studies in order to interpret data from non-validated studies, and in order to repeat the validation or update the model in other cohorts [44]. Some aspects of the study should be interpreted with care. First, the dataset did not include details on vascular involvement. A study of Satoi et al. and others showed that involvement of the portal vein was significantly associated with surgical unresectability, mainly based on the presence of occult metastases [27]. Second, serum CA19-9 had a high percentage of missing data and was only available as the highest value during the preoperative period. Nevertheless, imputing up to 50% of missing data is generally accepted, provided that the data are missing at random [45]. Also, there was no data on the number of PET-CT scans. Although PET-CT was not standard of care in the work-up and probably was rarely performed during the study period, this might have caused a selection bias. Lastly, the validation cohort was rather small, including only 60 events and might therefore lack power to identify model reproducibility. Additional validation in larger datasets is desired to provide further insights into the predictive value of the model.

In conclusion, based on this study, it is not yet possible to accurately predict occult metastases in patients with potentially resectable and borderline resectable pancreatic cancer. Diagnostic laparoscopy in selected patients identified 70% of occult metastases. Preoperative nutritional support, a low BMI, tumor size, solid tumor components, age, and indeterminate lesions on preoperative imaging were associated with occult metastases. Future studies should investigate biological markers added to preoperative clinical characteristics and should improve radiological parameters as potential predictors for occult metastases in order to develop a robust prediction model. Until then, performing a staging laparoscopy will remain mainly a choice of the multidisciplinary team meeting.

## Figures and Tables

**Table 1:** Baseline characteristics.

	Development Cohort n = 2262	Missing Values n (%)	Validation Cohort n = 663	Missing Values n (%)
Age, years (SD)	66 (10)	37 (2)	66 (10)	0 (0)
Male sex, n(%)	1213 (54)	32 (1)	361 (54)	0 (0)
BMI, kg/m^2^ (SD)	25.1 (4.3)	101 (5)	24.3 (3.7)	51 (8)
Any comorbidity, n(%)	1798 (80)	11 (1)	353 (54)	9 (1)
ECOG performance status, n(%) 0 1 ≥2	972 (47) 864 (42) 240 (12)	186 (8)	NA	NA
Weight loss, n(%)	1418 (74)	346 (15)	306 (47)	11 (2)
Preoperative biliary drainage, n(%)	956 (44)	102 (5)	304 (46)	0 (0)
Nutritional support, n (%) Oral Tube or TPN	794 (54) 157 (7)	88 (4)	NA 4 (1)	6 (1)
CA19-9, kU/l (IQR)	110 (20–490)	1003 (44)	102 (29–367)	143 (22)
Tumor location, n(%) Head/uncinate process Body/tail	1251 (81) 296 (19)	715 (32)	494 (75) 169 (25)	0 (0)
Tumor diameter, mm (IQR)	28 (21–37)	808 (36)	25 (20–34)	60 (9)
Cystic tumor composition, n(%)	230 (10)	47 (2)	99 (16)	27 (4)
Lymph node metastases, n(%)	322 (15)	105 (5)	NA	NA
Vascular involvement, n(%)	765 (35)	90 (4)	55 (8)	2 (0.3)
≥T3 tumor (TNM 7th edition), n(%)	332 (15)	106 (5)	NA	NA
Type of imaging, n(%) CT scan only MRI/MRCP only CT scan and MRI/MRCP		0 (0)		0 (0)
	1652 (73)		350 (53)	
	68 (3)		0 (0)	
	542 (24)		313 (47)	
Indeterminate lesions ^a^, n(%)	57 (3)	66 (3)	26 (4)	27 (4)
Weeks from imaging to surgery 0–2 2–4 4–6				
	583 (27)	129 (6)	NA	NA
	840 (39)			
	710 (33)			
Weeks from 1st consult to surgery (IQR)	4 (2–5)	75 (3)	6 (3–8)	181 (27)
DLS before exploration, n(%)	282 (13)	25 (1)	NA	NA

^a^ Subcentimeter or aspecific lesions on imaging, which could not be definitively characterized or excluded as metastases with further work-up. Abbreviations: BMI, body mass index; TPN, total parenteral feeding; TNM, tumor/node/metastasis classification of The American Joint Committee on Cancer; CT, computed tomography; MRI, magnetic resonance imaging; DLS, diagnostic laparoscopy; SD, standard deviation; IQR, interquartile range; NA, data not available from the validation cohort.

**Table 2:** Occult metastases.

	Development Cohort n = 235	Validation Cohort n = 60
Location, n (%) Liver Peritoneal Liver and peritoneal		
	143 (61)	39 (65)
	73 (31)	15 (25)
	19 (8)	6 (10)
Diagnosed with laparoscopy, n (%)	42 (18)	NA

Abbreviations: NA, data not available.

**Table 3:** Univariable analysis: predictors for occult metastases in the development cohort.

	No Occult Metastases n = 2027	Occult Metastases n = 235	*p*-Value
Age, years (SD)	66 (10)	68 (10)	0.014
Male sex, n (%)	1084 (54)	129 (56)	0.635
BMI, kg/m^2^ (SD)	25.1 (4.3)	24.6 (4.2)	0.082
Any comorbidity, n (%)	1608 (80)	190 (81)	0.758
ECOG performance status, n (%) 0 1 ≥2	888 (48) 757 (41) 212 (11)	84 (38) 107 (49) 28 (13)	0.028
Weight loss, n (%)	1255 (73)	163 (84)	0.002
Preoperative biliary drainage, n (%)	842 (44)	114 (50)	0.066
Nutritional support (tube or TPN), n (%)	128 (7)	29 (13)	0.001
CA19-9, kU/l (IQR)	103 (20–462)	206 (29–796)	0.016
Tumor location, n (%) Head/uncinate process Body/tail	1118 (81) 264 (19)	133 (81) 32 (19)	1.000
Tumor diameter, mm (IQR)	28 (21–36)	30 (25–40)	<0.001
Cystic tumor composition, n (%)	219 (11)	11 (5)	0.005
Lymph node metastases, n (%)	282 (15)	40 (18)	0.230
Vascular involvement, n (%)	672 (35)	93 (42)	0.034
≥T3 tumor (TNM 7th edition), n (%)	284 (15)	48 (22)	0.008
Indeterminate lesions ^a^, n (%)	40 (2)	17 (7.4)	<0.001
Weeks from imaging to surgery 0–2 2–4 4–6	522 (27) 749 (39) 636 (33)	61 (27) 91 (40) 74 (33)	0.959
Weeks from 1st consult to surgery (IQR)	3 (2–5)	3 (2–5)	0.009

^a^ Subcentimeter or aspecific lesions on imaging, which could not be definitively characterized or excluded as metastases with further work-up. Abbreviations: SD, standard deviation; BMI, body mass index; ECOG, Eastern Cooperative Oncology Group; TPN, total parenteral nutrition; IQR, inter quartile range; TNM, tumor/node/metastasis classification of The American Joint Committee on Cancer.

**Table 4:** Final model of predictors for occult metastases in the development cohort.

Variables	β Coefficient	OR (95%CI)	*p*-Value
Intercept	−4.009		
Age	0.018	1.02 (1.00–1.03)	0.02
BMI	−0.039	0.96 (0.93–1.00)	0.04
Nutritional support	0.549	1.73 (1.10–2.74)	0.02
Solid tumor composition (versus cystic)	−0.834	2.33 (1.20–4.35)	0.01
Tumor diameter (log)	0.468	1.60 (1.04–2.45)	0.04
Indeterminate lesions on imaging ^a^	1.387	4.01 (2.16–7.43)	<0.001

^a^ Subcentimeter or aspecific lesions on imaging, which could not be definitively characterized or excluded as metastases with further work-up. Abbreviations: BMI, body mass index.

## Data Availability

The data presented in this study are available on request from the corresponding author.
